# Uncovering the hidden biology of fibrinaloid microclot complexes in complex, inflammatory diseases

**DOI:** 10.1017/qrd.2026.10022

**Published:** 2026-07-31

**Authors:** Justine Grixti, Etheresia Pretorius, Douglas B. Kell

**Affiliations:** 1 Department of Biochemistry, Cell and Systems Biology, Institute of Systems, Molecular and Integrative Biology, University of Liverpool, UK; 2 Physiological Sciences, Stellenbosch University, South Africa

**Keywords:** amyloid, inflammation, microcirculation, microclots, oxidative stress

## Abstract

Blood can clot into anomalous, fibrinolysis-resistant forms that arise from prothrombotic seeding areas, including damaged cellular debris and membrane-derived surfaces, giving rise to what we have termed fibrinaloid microclot complexes (colloquially: microclots). Their proteolytic resistance is due in part to the fact that they are amyloid in nature, and they can also entrap inhibitors of proteolysis. They consist of a variety of proteins besides the expected fibrin, and are highly enriched for other amyloidogenic proteins (in contrast to normal clots, whose proteome largely reflects the soluble plasma proteome). They also contain DNA in the form of neutrophil extracellular traps (NETs). Importantly, fibrinaloid microclot complexes are heterogeneous structures comprising multiple phenotypic forms, including those that nucleate and grow on cellular debris such as damaged membranes, microparticles, and immune-derived material. We consider that these debris-associated complexes can act as catalytic scaffolds that recruit fibrin(ogen) and inflammatory molecules, thereby amplifying amyloidogenic transformation and prothrombotic activity. Fibrinaloid microclot complexes have been reported in a widening range of chronic inflammatory and thrombo-inflammatory diseases in which they have been sought, and are highly enriched for amyloidogenic proteins. Additionally, the thrombi extracted from ischaemic stroke also contain proteins in an amyloid form. One mechanism that explains how such macroclots can form and block arteries larger than any leading to them is that this occurs via the accretion of microclots that already contain amyloid. We here show that these microclots exhibit a classical ‘apple-green’ birefringence when stained with the dye Congo red. It is now important to determine whether inhibiting amyloid-forming clot transitions has therapeutic value.

## Graphical abstract

**Figure:**
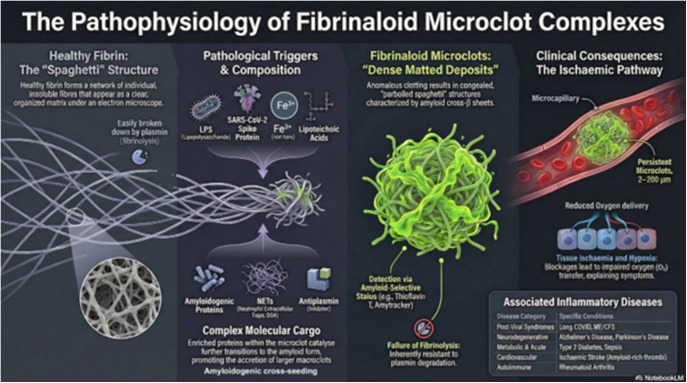

## Introduction

Blood clotting is traditionally understood as a thrombin-driven process in which fibrin polymers form structured networks that are susceptible to regulated fibrinolysis; however, under inflammatory conditions, clotting can also originate from prothrombotic seeding areas that give rise to structurally distinct, fibrinolysis-resistant assemblies. In healthy haemostasis, these fibres form branching networks whose architecture is influenced by fibrinogen concentration, thrombin generation, flow, and cross-linking. In the electron microscope, these appear like spaghetti (Weisel and Litvinov, 2017; Pretorius *et al.*, 2018b).

However, blood can also clot into anomalous forms that are significantly resistant to fibrinolysis and that in the electron microscope appear instead as ‘dense matted deposits’ that take on the appearance of parboiled spaghetti that has been allowed to congeal (e.g., (Pretorius *et al.*, 2010; Pretorius *et al.*, 2013a; Swanepoel *et al.*, 2016).

Based on staining using a variety of established amyloid dyes, this form was seen as being consistent with these structures being amyloid in nature (Pretorius *et al.*, 2016b), involving the kind of cross-*β* sheets seen in prion diseases and classical amyloidoses (Kell and Pretorius, 2017). These observations suggest that pathological clotting does not simply reflect dysregulated thrombin activity; one mechanism is that of surface-driven nucleation processes in which damaged biological interfaces act as catalytic scaffolds for amyloidogenic fibrin(ogen) assembly. We have referred to these microclot complexes as fibrinaloid, and they typically possess a diameter in the range 2–200 μm. They also contain neutrophil extracellular traps (NETs (Thierry *et al.*, 2025). Importantly, these complexes represent heterogeneous phenotypic forms, reflecting their origins on distinct seeding substrates, such as membrane debris, microparticles, NET-derived nucleoprotein scaffolds, and plasma protein aggregates.

**Figure 1.**
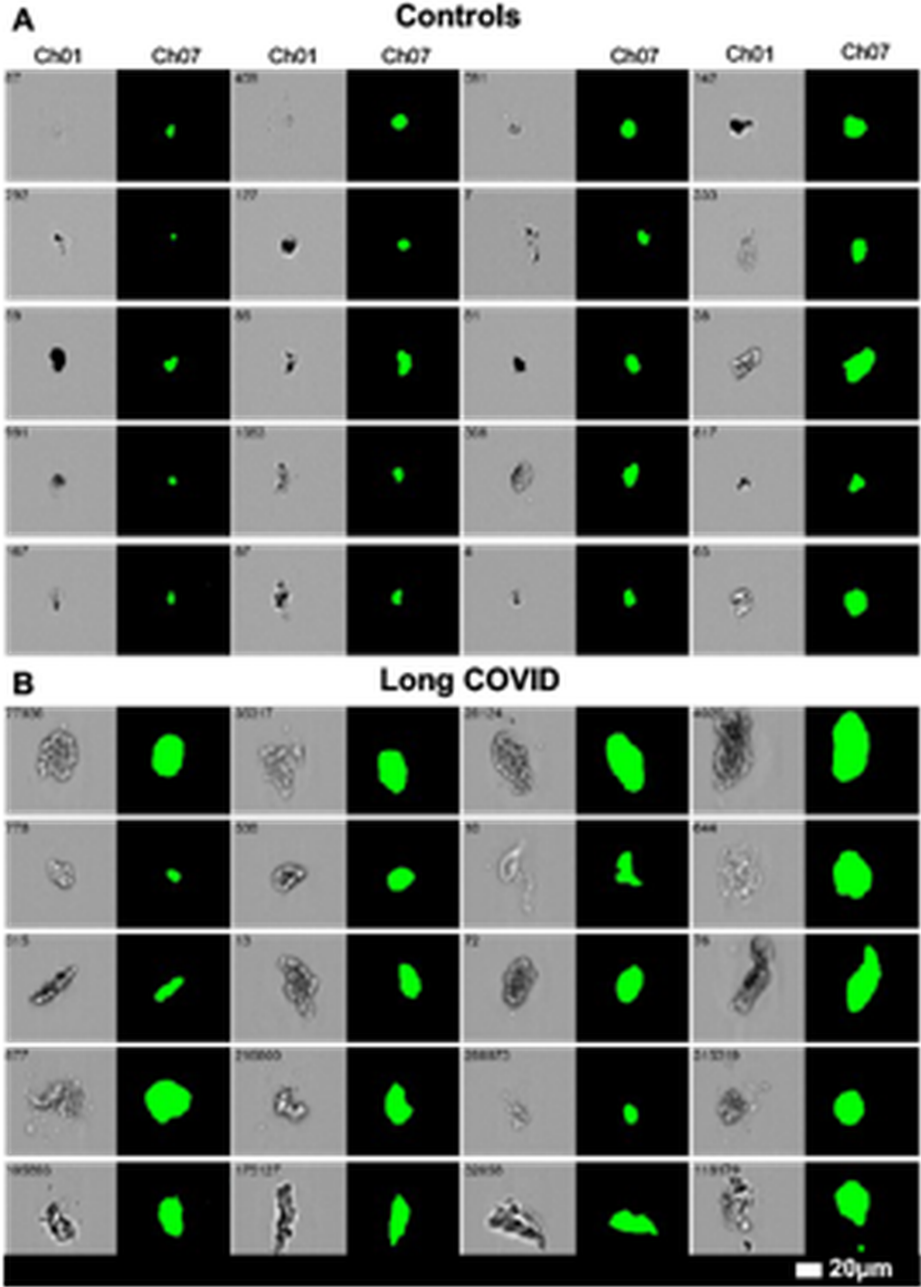
Fibrinaloid microclot complexes stained with Thioflavin T (ThT) as detected in platelet-poor plasma (PPP) using imaging flow cytometry. Image taken from the CC-BY 4.0 publication (Turner *et al.*, 2023b): (a) Controls and (b) Long Covid. Figure 1. long description.

The fibrinaloid microclot complexes are typically observed using fluorogenic amyloid stains such as thioflavin T (Biancalana and Koide, 2010; Gade Malmos *et al.*, 2017; Xue *et al.*, 2017) or the oligothiophene ‘Amytracker’ dyes (de Waal *et al.*, 2018; Pretorius *et al.*, 2017b, 2018a). As is well known, amyloids are much more resistant to normal proteolysis than the soluble forms formed at the ribosome. Figure 1 shows examples of representative microclot complexes stained with Thioflavin T and detected with imaging flow cytometry. Together, these findings are considered to define a mechanistically distinct mode of clot formation in which smaller amyloidogenic, surface-seeded complexes can precede and potentially drive the development of larger, clinically relevant thrombotic structures, as is common in classical amyloidoses.

Fibrinaloid microclot complexes have now been reported across a widening range of inflammatory and thrombo-inflammatory conditions (Kell and Pretorius, 2018a) (Table 1), including acute COVID-19, Long COVID, sepsis, type 2 diabetes, Parkinson’s disease, rheumatoid arthritis, and Alzheimer-related syndromes, with support coming both from our own studies and from independent groups. However, while the presence of fibrinaloid microclot complexes has been observed across these diverse conditions, detailed mechanistic characterization of their phenotypes and their origins from specific prothrombotic seeding areas has not been uniformly established.

**Table 1.** Summary of representative evidence for fibrinaloid microclot complexes across selected disease settings Table 1. long description.

Condition	Representative independent studies	Representative Kell/Pretorius studies	Sample/material	Readout	Amyloid-selective stain	Proteomics	NET association	Clinical link
Acute COVID-19	Bunch *et al.* (2022)	Grobler *et al.* (2020), Pretorius *et al.* (2020)	PPP/plasma	Microscopy/staining	Yes	Limited	Variable	Coagulopathy
Long COVID	Dalton *et al.* (2024), Okuducu *et al.* (2024), Irimia *et al.* (2025), Steifman *et al.* (2026)	Booyens *et al.* (2025), Kell *et al.* (2022), Kell and Pretorius (2022, 2023), Kruger *et al.* (2025), Kruger *et al.* (2022), Nunes *et al.* (2026), Pretorius *et al.* (2022), Pretorius *et al.* (2021), Thierry *et al.* (2025), Turner *et al.* (2023a), Turner *et al.* (2023b), Turner *et al.* (2024)	PPP/plasma	IFC/microscopy/proteomics	Yes	Yes	Yes	Symptom burden
Sepsis	Schofield *et al.* (2024)	Presaged in Kell and Pretorius (2018b)	Plasma	Amyloid-fibrinogen aggregates	Yes	No	Not central	DIC/mortality
Stroke thrombi	–	Grixti *et al.* (2024a), Grixti *et al.* (2025)	Retrieved thrombi	Amyloid staining/proteomics	Yes	Yes	Not central	Fibrinolysis resistance
T2D	–	de Waal *et al.* (2018), Pretorius and Bester (2016), Pretorius *et al.* (2015), Pretorius *et al.* (2017b), Pretorius *et al.* (2020), Soma and Pretorius (2015)	PPP/fibrin	Microscopy/stains	Yes	Limited	No	Abnormal clotting
Parkinson’s/Alzheimer’s	–	Adams *et al.* (2019), de Waal *et al.* (2018), Grobler *et al.* (2023), Itzhaki *et al.* (2016), Pretorius *et al.* (2016a), Pretorius *et al.* (2018b) and van Vuuren *et al.* (2020)	Plasma/fibrin	Correlative LM/EM, stains	Yes	Limited	No	Chronic inflammation
Rheumatoid arthritis	–	Pretorius *et al.* (2017a)	Plasma/fibrin	EM	No	No	No	Chronic inflammation
ME/CFS		Kell and Pretorius (2022), Nunes *et al.* (2022), Nunes *et al.* (2023), Nunes *et al.* (2026)	Plasma/fibrin	Microscopy /stains	Yes	Yes	No	Chronic inflammation

The table distinguishes disease context, study provenance, material analysed, analytical modality, and the type of supporting evidence. It is intended as an evidence map rather than an exhaustive systematic review.

To date, such phenotypic resolution, including the identification of distinct seeding substrates such as cellular debris, microparticles, and nucleoprotein complexes, has been most clearly demonstrated in COVID-19, Long COVID, and type 2 diabetes. In contrast, in other disease settings, fibrinaloid microclots have largely been described at the level of presence, morphology, or proteomic enrichment, without explicit discrimination of their underlying phenotypic diversity or seeding mechanisms.

The thrombi causing ischaemic stroke also display amyloid (Grixti *et al.*, 2024a, 2025), and the fibrinaloid microclots can be formed using just purified fibrinogen, thrombin, and a suitable trigger molecule (Grixti *et al.*, 2024a; Pretorius *et al.*, 2016b).

Several triggers of amyloidogenic clotting have been observed experimentally, including ferric ions (Pretorius *et al.*, 2013a, 2013b, 2014; Pretorius and Kell, 2014), bacterial lipopolysaccharide (LPS) (Pretorius *et al.*, 2016b), lipoteichoic acids (Pretorius *et al.*, 2018a), and the SARS-CoV-2 spike protein (Grobbelaar *et al.*, 2021. These have all been found to trigger the amyloidogenic clotting, often at minuscule levels (Pretorius *et al.*, 2016b).

The SARS-CoV-2 spike protein is itself highly amyloidogenic (Nyström and Hammarström, 2022; Westman *et al.*, 2025), its amyloidogenicity (in different strains of SARS-CoV-2) being related to the virulence of the strains (Grobbelaar *et al.*, 2022). Taken together, these findings are consistent with the view that clot amyloidogenesis can lie on an important disease pathway.

It is recognised that thioflavin T, while seen as a gold standard amyloid stain (Biancalana *et al.*, 2009; Xue *et al.*, 2017), is not entirely specific for amyloid (Thierry *et al.*, 2025). To this end, we have also employed the more selective oligothiophene ‘Amytracker’ dyes, again showing that they stained fibrinaloid microclot complexes (e.g., see de Waal *et al.*, 2018; Pretorius *et al.*, 2017b, 2017c, 2018a). Another ‘classical’ amyloid dye is Congo Red, which shows an ‘apple-green’ birefringence when illuminated with polarised light (e.g., see Elghetany *et al.*, 1989; Howie, 2015; Yakupova *et al.*, 2019; cf. Howie, 2024). We here show, for the first time, fibrinaloid microclots assessed in this way with Congo Red.

**Figure 2.**
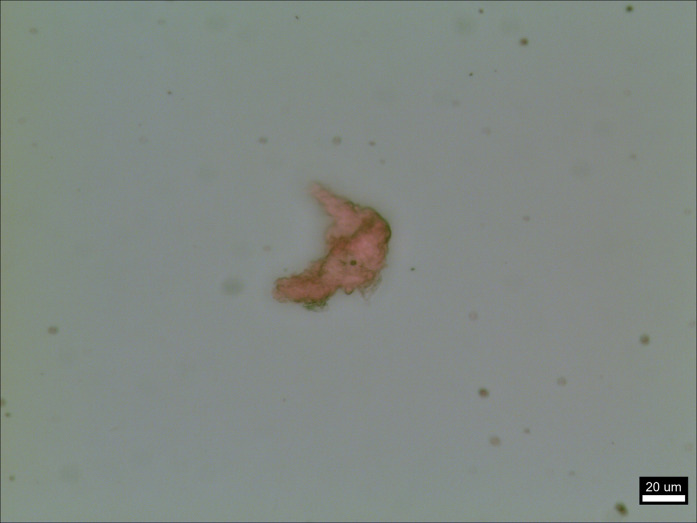
A fibrinaloid microclot created using purified fibrinogen and thrombin as in Methods, stained with 0.3 mM Congo Red, and imaged in a polarising microscope as in Methods. The classical ‘apple-green’ birefringence is observed in particular around the periphery of the microclot.

Figure 2 shows images of a fibrinaloid microclot created *in vitro* (as per (Grixti *et al.*, 2024b; Pretorius *et al.*, 2016b)), stained with the classical amyloid dye Congo Red (Dapson, 2018) and assessed using a polarising microscope as described in Methods, below. It displays the classical ‘apple green’ birefringence, providing further evidence for the amyloid nature of the microclots.

In Long COVID, increased FMC burdens have been reported in platelet-poor plasma by more than one group (Dalton *et al.*, 2024; Okuducu *et al.*, 2024; Irimia *et al.*, 2025; Steifman *et al.*, 2026), although with inter-individual variability and continuing debate over assay standardisation and interpretation. These observations are consistent with the broader concept that persistent thrombo-inflammation, endothelial activation, and impaired fibrinolysis may contribute to symptom burden in at least a subset of patients. Within this framework, microcirculatory impairment provides one plausible route by which diverse symptoms may arise, although the degree of causal contribution is still being defined.

The effect of these insoluble FMCs is potentially to partially block or interfere with blood flow in microcapillaries (the ‘microcirculation’) and hence O_2_ transfer to tissues. Taking Long COVID as an example, such a blockage can straightforwardly and self-consistently explain a great many symptoms that accompany this highly variable syndrome, including fatigue (Kell *et al.*, 2022), post-exertional malaise (Kell and Pretorius, 2022), autoimmunity (Kell and Pretorius, 2023), atrial fibrillation (Kell *et al.*, 2024b), fibromyalgia (Kell and Pretorius, 2026), POTS (Kell *et al.*, 2024a), and tinnitus (Kell and Pretorius, 2025a). This said, we do recognise that other mechanisms are also involved.

Emerging evidence also indicates that FMCs represent a spectrum of structurally and mechanistically distinct entities rather than a single homogeneous clotting product. While earlier work has focused primarily on fibrin(ogen)-derived amyloid formation, it is now clear that a substantial proportion of these complexes originate from prothrombotic seeding areas, including cellular debris, membrane fragments, microparticles, and nucleoprotein immune structures. These debris-associated complexes are fundamentally different from classical fibrin networks in that they do not form through thrombin-driven polymerisation, do not integrate into organised fibrin fibre architectures, and are largely resistant to conventional plasmin-mediated fibrinolysis. In parallel, a related but distinct process occurs in which soluble inflammatory and amyloidogenic molecules interact with fibrinogen and, upon thrombin activation, become incorporated into fibrin networks, giving rise to the dense matted deposits previously described. These observations support the existence of at least two overlapping but mechanistically distinct pathways: (i) surface-seeded formation of debris-associated fibrinaloid complexes, and (ii) thrombin-mediated formation of structurally altered fibrin networks incorporating inflammatory components. This distinction is critical, as conventional coagulation and fibrinolysis assays are likely to capture only the latter, thereby underestimating or entirely missing the presence of debris-associated fibrinaloid structures and contributing to ongoing discrepancies in the literature.

**Figure 3.**
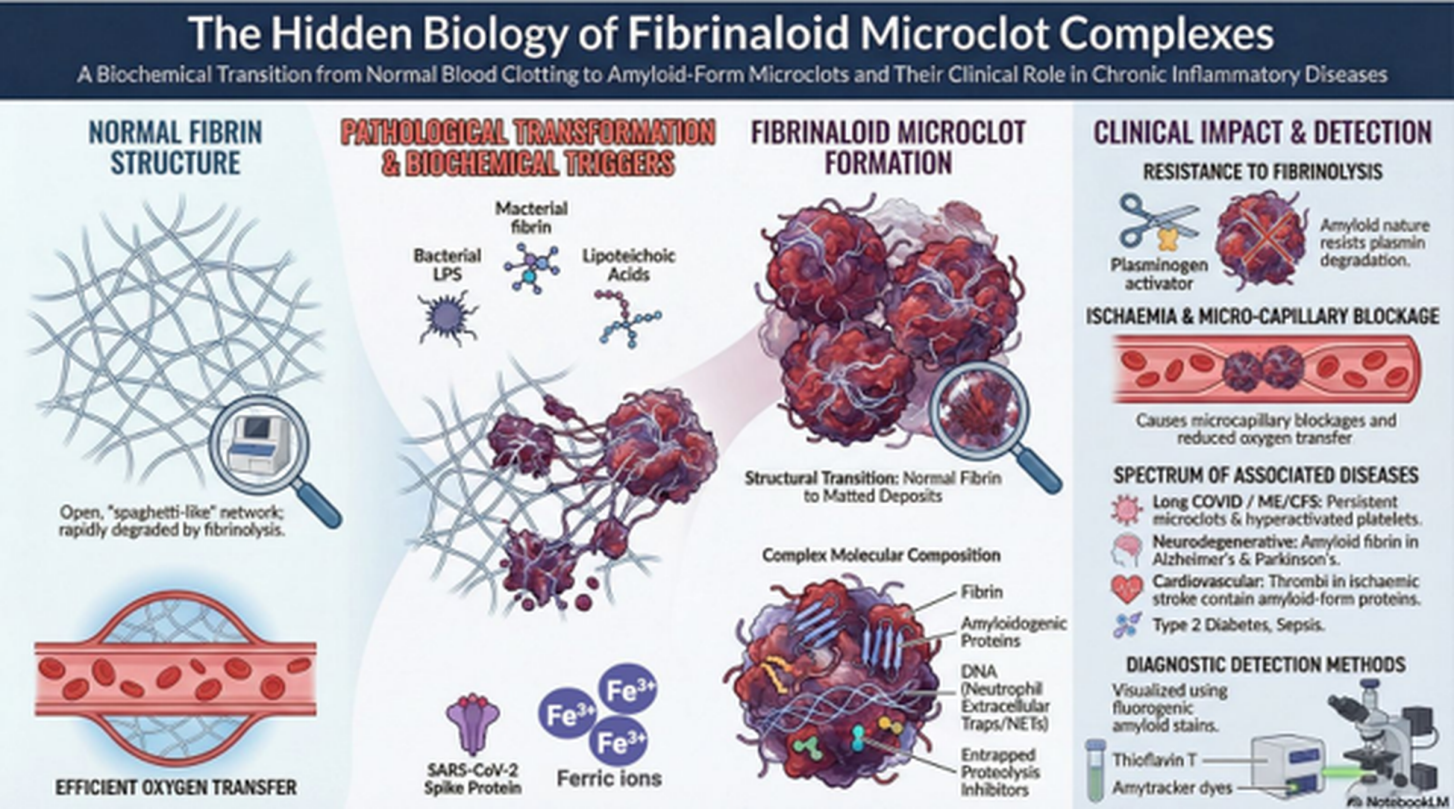
A summary of fibrinaloid microclot complexes. This figure (and also the graphical abstract) was generated on 23 April 2026 with the assistance of the artificial intelligence tool NotebookLM (https://notebooklm.google/), using author-provided text and figure concepts; all scientific content, interpretation, and final design decisions were verified and approved by the authors. Figure 3. long description.

We have used the Amylogram programme (Burdukiewicz *et al.*, 2017; Szulc *et al.*, 2021) to assess the amyloidogenicity of individual proteins (the output is a score between 0 and 1, with anything over ca 0.75 being significantly and experimentally amyloidogenic (Kell *et al.*, 2025). The proteome of normal thrombi (Ząbczyk *et al.*, 2019) largely reflects that of the soluble plasma proteome. However, the proteomes of the various fibrinaloid microclot complexes are very different, with the proteins that are enriched in them being themselves highly amyloidogenic (Kell *et al.*, 2025; Kell and Pretorius, 2024, 2025b). Table 2 shows proteins enriched in the microclots, a dataset (Kell and Pretorius, 2024) to which we have added their Amylogram scores. Each of these exceeds 0.75, some by a considerable margin. One explanation is simply that this is caused by cross-seeding of these amyloidogenic proteins catalysing the transition to the amyloid form, and that the macroclots in ischaemic stroke and other cardiovascular diseases can form via accretion of these microclots. Of course, their amyloid nature again straightforwardly explains their resistance to the normal fibrinolysis that would be catalysed by plasmin. A pictorial summary is given in Figure 3.

**Table 2.** Proteins enriched or lowered in fibrinaloid microclots relative to normal clots (Kell and Pretorius, 2024), with their Amylogram scores Table 2. long description.

Protein	Uniprot ID	Amylogram score
Adiponectin	Q15848	0.833
Kallikrein	P03952	0.769
LBLC1/BNIB1/BNIFB1/LPLUNC1	Q8TDL5	0.918
Platelet factor 4	P02776	0.778
Periostin	Q15063	0.914
Thrombospondin-1	P07996	0.863

Although the broader disease-spanning framework has been developed largely through our own work, recent reports from independent groups in sepsis and Long COVID indicate that the phenomenon is reproducible beyond a single laboratory context. At the same time, the field would benefit greatly from further methodological standardisation, head-to-head comparison of assays, and additional external replication across disease settings.

We note that treatments, such as nattokinase and lumbricase, that serve to remove microclots can be efficacious (Eckey *et al.*, 2025). Consequently, our next strategy is to develop methods that are not simply anticoagulating (though such can be efficacious (Wright *et al.*, 2025)), but that can inhibit the transition of fibrin(ogen) to the amyloid form. Success here would add considerably to the existing evidence (such as that relating amyloidogenicity to virulence (Grobbelaar *et al.*, 2021, 2022) that the microclots are not only present but on the disease pathway.

## Methods

To create and analyse fibrinaloid microclots with Congo Red *in vitro* , 30 ng/mL of bacterial LPS were added to purified FBG at 2.6 mg/mL. To this, Congo Red was added at 0.3 mM, followed by 7 U of Thrombin. All concentrations stated are final. Images were taken on an Axio Observer.Z1 (Zeiss, Jena, Germany) microscope using a halogen lamp (HAL100) and a polariser filter with a GXCAM-3 (GT Vision, Wickhambrook, UK). Images were acquired with the software GXCam version 7.3. All images were taken with a Plan-APOCHROMAT FLUAR 20 × 0.75 DIC and saved in .tif format. The methods and data for Figure 1 were obtained using imaging flow cytometry, the image being taken from a CC-BY 4.0 publication (Turner *et al.*, 2023b).

## Conclusion

FMCs have now been reported across a widening range of inflammatory and thrombo-inflammatory conditions (Kell and Pretorius, 2018a) (Table 1), including acute COVID-19, Long COVID, sepsis, type 2 diabetes, Parkinson’s disease, rheumatoid arthritis, and Alzheimer-related syndromes, with support coming both from our own studies and from independent groups. However, while the presence of fibrinaloid microclot complexes has been observed across these diverse conditions, detailed mechanistic characterization of their phenotypes and their origins from specific prothrombotic seeding areas has not been uniformly established, although our group has identified the need for better phenotyping (Nunes *et al.*, 2026). To date, such phenotypic resolution, including the identification of distinct seeding substrates such as cellular debris, microparticles, and nucleoprotein complexes, has been most clearly demonstrated in COVID-19, Long COVID, and type 2 diabetes. In contrast, in other disease settings, fibrinaloid microclots have largely been described at the level of presence, morphology, or proteomic enrichment, without explicit discrimination of their underlying phenotypic diversity or seeding mechanisms.

This distinction is critical, as it indicates that FMCs are not a single pathological entity but a spectrum of mechanistically distinct, surface-seeded assemblies, and that resolving their phenotypic diversity and seeding origins is essential for understanding their role in disease pathogenesis, as well as for developing targeted diagnostic and therapeutic strategies.

## Long descriptions

### Figure 1. Long description

The photo is divided into two horizontal sections, A and B, each containing a grid of microscopic images.

* Section A is titled Controls. It consists of five rows and eight columns. The columns alternate between C h 01, which shows brightfield grayscale images of small particles, and C h 07, which shows corresponding fluorescent green signals from Th T staining. The particles in this section are generally small, sparse, and appear as tiny dark specks in brightfield and small green dots in fluorescence.

* Section B is titled Long C O V I D. It follows the same five-row, eight-column alternating format of Ch 01 and Ch 07. In contrast to the controls, the microclot complexes here are significantly larger, more numerous, and exhibit irregular, fibrous shapes. The green fluorescent signals in the C h 07 columns are much brighter and cover larger surface areas, indicating a higher concentration of amyloid-like protein structures.

* A scale bar at the bottom right indicates 20 micrometers. Each individual image frame contains a small identification number in the top-left corner.

Navigate back to Figure 1..

### Table 1. Long description

The table consists of nine columns: Condition, Representative independent studies, Representative Kell/Pretorius studies, Sample/material, Readout, Amyloid-selective stain, Proteomics, NET association, and Clinical link.

* Acute COVID-19: Studies by Bunch et al., Grobler et al., and Pretorius et al. using P P P or plasma. Readout via microscopy/staining. Amyloid stain: Yes. Proteomics: Limited. NET association: Variable. Clinical link: Coagulopathy.

* Long COVID: Extensive studies including Dalton et al., Okuducu et al., and numerous Kell/Pretorius studies. Sample: PPP or plasma. Readout: IF C, microscopy, and proteomics. Amyloid stain: Yes. Proteomics: Yes. NET association: Yes. Clinical link: Symptom burden.

* Sepsis: Studies by Schofield et al. and Kell/Pretorius. Sample: Plasma. Readout: Amyloid-fibrinogen aggregates. Amyloid stain: Yes. Proteomics: No. NET association: Not central. Clinical link: D I C or mortality.

* Stroke thrombi: Kell/Pretorius studies (Grixti et al.). Sample: Retrieved thrombi. Readout: Amyloid staining and proteomics. Amyloid stain: Yes. Proteomics: Yes. NET association: Not central. Clinical link: Fibrinolysis resistance.

* T2 D: Kell/Pretorius studies. Sample: PP P or fibrin. Readout: Microscopy/stains. Amyloid stain: Yes. Proteomics: Limited. NET association: No. Clinical link: Abnormal clotting.

* Parkinson’s/Alzheimer’s: Kell/Pretorius studies. Sample: Plasma or fibrin. Readout: Correlative L M or E M and stains. Amyloid stain: Yes. Proteomics: Limited. N E T association: No. Clinical link: Chronic inflammation.

* Rheumatoid arthritis: Kell/Pretorius studies. Sample: Plasma or fibrin. Readout: E M. Amyloid stain: No. Proteomics: No. N E T association: No. Clinical link: Chronic inflammation.

* ME/CFS: Kell/Pretorius studies (Nunes et al.). Sample: Plasma or fibrin. Readout: Microscopy/stains. Amyloid stain: Yes. Proteomics: Yes. NET association: No. Clinical link: Chronic inflammation.

Navigate back to Table 1..

### Table 2. Long description

The table consists of three columns: Protein, UNIPROT ID, and Amylogram score.

* Adiponectin: UNIPROT ID Q1 58 48, Amylogram score 0.833.

* Kallikrein: UNIPROT ID P03952, Amylogram score 0.769.

* LBLC1/BNIB1/BNIFB1/LPLUNC1: UNIPROT ID Q8TDL5, Amylogram score 0.918.

* Platelet factor 4: UNIPROT ID P02776, Amylogram score 0.778.

* Periostin: UNIPROT ID Q15063, Amylogram score 0.914.

* Thrombospondin-1: UNIPROT ID P07996, Amylogram score 0.863.

Navigate back to Table 2..

### Figure 3. Long description

Panel 1, Normal Fibrin Structure. Top shows an open spaghetti-like network of thin blue fibers. A magnifying glass highlights the porous nature. Bottom shows a cross-section of a blood vessel with red blood cells flowing freely, labeled Efficient Oxygen Transfer.

Panel 2, Pathological Transformation and Biochemical Triggers. Triggers include Bacterial L P S, Macterial fibrin, Lipoteichoic Acids, SARS-CoV-2 Spike Protein, and Ferric ions (F e 3 super plus). These triggers interact with the fibrin network, causing it to collapse into dense, dark red matted deposits.

Panel 3, Fibrinaloid Microclot Formation. Top shows a cluster of large, dense red microclots. A magnifying glass shows the structural transition from normal fibrin to matted deposits. Bottom shows a Complex Molecular Composition diagram of a single microclot containing Fibrin, Amyloidogenic Proteins, DNA (Neutrophil Extracellular Traps or NETs), and Entrapped Proteolysis Inhibitors.

Panel 4, Clinical Impact and Detection. Top section, Resistance to Fibrinolysis, shows a pair of scissors (Plasminogen activator) unable to cut a microclot due to its amyloid nature. Middle section, Ischaemia and Micro-capillary Blockage, shows microclots obstructing a narrow vessel, reducing oxygen transfer. Bottom section lists associated diseases: Long COVID / ME/CF S, Neurodegenerative (Alzheimer’s and Parkinson’s), Cardiovascular (Ischaemic stroke), and Type 2 Diabetes or Sepsis. The final section, Diagnostic Detection Methods, shows a microscope setup using Thioflavin T and Amytracker dyes.

Navigate back to Figure 3..
