# Supplementary material for: Uncovering the hidden biology of fibrinaloid microclot complexes in complex, inflammatory diseases
Source: QRB Discov. 2026 Jul 31;7:e9. doi: 10.1017/qrd.2026.10022 (PMC13434857; doi:10.1017/qrd.2026.10022)
Supplement: Peer review history

# Editor report: Recommendation: Uncovering the hidden biology of fibrinaloid microclot complexes in complex, inflammatory diseases — R0/PR2

**DOI:** 10.1017/qrd.2026.10022.pr2

**Affiliations:** Director of the Kavli Institute for Nanoscience Discovery at Oxford, University of Oxford, United Kingdom of Great Britain and Northern Ireland

See notes on article for reviewing history

# Editor report: Decision: Uncovering the hidden biology of fibrinaloid microclot complexes in complex, inflammatory diseases — R0/PR3

**DOI:** 10.1017/qrd.2026.10022.pr3

**Affiliations:** Director of the Kavli Institute for Nanoscience Discovery at Oxford, University of Oxford, United Kingdom of Great Britain and Northern Ireland

No accompanying comment.
